# Association of CNR1 gene and cannabinoid 1 receptor protein in the human brain

**DOI:** 10.1002/jnr.25149

**Published:** 2022-11-28

**Authors:** Kyoungjune Pak, Tatu Kantonen, Laura Pekkarinen, Pirjo Nuutila, Lauri Nummenmaa

**Affiliations:** ^1^ Turku PET Centre University of Turku Turku Finland; ^2^ Turku University Hospital Turku Finland; ^3^ Department of Nuclear Medicine and Biomedical Research Institute Pusan National University Hospital Busan Republic of Korea; ^4^ Clinical Neurosciences University of Turku Turku Finland; ^5^ Department of Endocrinology Turku University Hospital Turku Finland; ^6^ Department of Psychology University of Turku Turku Finland

**Keywords:** cannabinoid receptor, messenger RNA, positron emission tomography

## Abstract

We aimed to integrate genomic mapping from brain mRNA atlas with the protein expression from positron emission tomography (PET) scans of type 1 cannabinoid (CB1) receptor and to compare the predictive power of CB1 receptor with those of other neuroreceptor/transporters using a meta‐analysis. Volume of distribution (*V*_*T*_) from F18‐FMPEP‐d2 PET scans, CNR1 gene (Cannabinoid receptor 1) expression, and H3‐CP55940 binding were calculated and correlation analysis was performed. Between *V*_*T*_ of F18‐FMPEP‐d2 PET scans and CNR1 mRNA expression, moderate strength of correlation was observed (*rho* = .5067, *p* = .0337). Strong positive correlation was also found between CNR1 mRNA expression and H3‐CP55940 binding (*r* = .6336, *p* = .0364), validating the finding between F18‐FMPEP‐d2 PET scans and CNR1 mRNA. The correlation between *V*_*T*_ of F18‐FMPEP‐d2 PET scans and H3‐CP55940 binding was marginally significant (*r* = .5025, *p* = .0563). From the meta‐analysis, the correlation coefficient between mRNA expression and protein expressions ranged from −.10 to .99, with a pooled effect of .76. In conclusion, we observed the moderate to strong associations between gene and protein expression for CB1 receptor in the human brain, which was validated by autoradiography. We combined the autoradiographic finding with PET of CB1 receptor, producing the density atlas map of CB1 receptor. From the meta‐analysis, the moderate to strong correlation was observed between mRNA expression and protein expressions across multiple genes. Further study is needed to investigate the relationship between multiple genes and in vivo proteins to improve and accelerate drug development.

## Graphical abstract

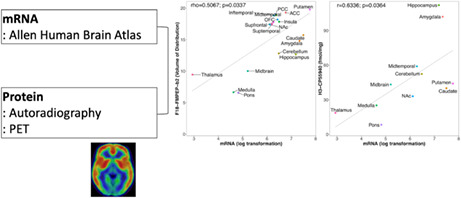

**Significance**

The moderate to strong associations between gene and protein expression for CB1 receptor in the human brain were observed and we produced the density atlas map of CB1 receptor for the first time in the human brain. From the meta‐analysis, the moderate to strong correlation was observed between mRNA expression and protein expressions across multiple genes.

## 1 INTRODUCTION

The use of cannabinoids is of growing interest since several studies supported a great variety of pharmacological properties that would be useful in different pathologies, including neurological and psychiatric disorders (Black et al., 2019). In 1985, the synthetic tetrahydrocannabinol (THC) derivatives dronabinol and nabilone are the first cannabinoids approved by the U.S. Food and Drug Administration (FDA) for the treatment of acquired immunodeficiency syndrome‐induced anorexia (dronabinol) and chemotherapy‐induced nausea and vomiting (dronabinol and nabilone) (Khalsa et al., 2022). Cannbidiol (CBD) was approved in 2018 by FDA after it was shown to be effective and safe for treating seizures associated with Lennox–Gastaut syndrome or Dravet syndrome in patients aged 2 years and older (Khalsa et al., 2022). An additional cannabis‐based drug is nabiximols, a combination of THC and CBD, which was approved for the treatment of spasticity in multiple sclerosis (Khalsa et al., 2022). The endocannabinoid system consists of type 1 (CB1) and type 2 (CB2) cannabinoid receptors, endogenous ligands, and their metabolic enzymes (Tao et al., 2020). CB1 receptor, encoded by CNR1 gene, is expressed in cortex, hippocampus, amygdala, basal ganglia, substantia nigra, and cerebellum (Gifford et al., 2002; Herkenham et al., 1990; Mackie, 2005; Westlake et al., 1994). CB1 receptors are found primarily in the presynapses of the neurons (Mechoulam & Parker, 2013). They act as neuromodulators, inhibiting the release of neurotransmitters like gamma‐aminobutyric acid, glutamate, and dopamine into the synapse (Dickens et al., 2020; Piomelli, 2003). Alterations of CB1 receptor availability have been reported in neuropsychiatry disorders, such as post‐traumatic stress disorder and schizophrenia (Neumeister et al., 2013; Ranganathan et al., 2016; Volk et al., 2014). In addition, CB1 receptor has an important role in the regulation of emotional behavior and cognitive function, such as anxiety, fear, and memory (Häring et al., 2015). Also, CB1 receptor antagonist, rimonabant was introduced as an anti‐obesity drug, however, was withdrawn due to serious side effects including depressive disorders and suicide (Christensen et al., 2007; Di Marzo & Despres, 2009; Faulconbridge et al., 2009; Thomas et al., 2014). Therefore, CB1 receptor has been a target for drug development and in vivo imaging biomarker for neuropsychiatric disorders (Van Laere, 2007).

The Allen Human Brain Atlas is a freely available multimodal atlas of gene expression with visualization and data‐mining resources that comprises a comprehensive array‐based dataset of gene expression (Shen et al., 2012). With the help of the Allen Human Brain Atlas, genomic maps visualize not only the gene expression across whole brain regions, but also the functional profile of brain structures (Sandberg et al., 2000). Previous studies have proved the predictive power of brain mRNA mapping for in vivo protein expression from positron emission tomography (PET) for serotonin receptors (Beliveau et al., 2017; Komorowski et al., 2017; Rizzo et al., 2014), opioid receptors (Rizzo et al., 2014), and monoamine oxidase A (Komorowski et al., 2017; Zanotti‐Fregonara et al., 2014), based on the key assumption that mRNA expression predicts protein expression. However, the studies on serotonin transporter or dopamine transporter showed a weak association between mRNA and protein levels, emphasizing the role of translational and post‐translational mechanisms (Beliveau et al., 2017; Pak et al., 2022). To date, there have been no published data on the association of CB1 gene expression with CB1 protein expression in humans. Previous studies of CB1 receptor have focused mainly on receptor binding, signal transduction, with lack of knowledge on CB1 gene regulation (Laprairie et al., 2012). Pharmacological exposures such as methamphetamine, alcohols, cannabinoids as well as physiological processes are known to modulate CB1 receptor mRNA expression (Laprairie et al., 2012). Therefore, it is more timely than ever to integrate genomic mapping from brain mRNA atlas with the protein expression from PET scans for better understanding of CB1 receptor of the human brain (Gryglewski et al., 2018). Here, we investigated the association between CNR1 gene expression from the Allen Human Brain Atlas and CB1 receptor expression from F18‐FMPEP‐d2 PET scans. In addition to protein expressions from PET scans, H3‐CP55940 bindings from autoradiography (Glass et al., 1997) were collected, and analyzed to validate the findings of this study. Finally, to compare the predictive power of CB1 receptor with those of other neuroreceptor/transporters, we conducted a meta‐analysis on the association between mRNA expression from the Allen Human Brain Atlas and protein expressions from multi‐tracer scans.

## 2 MATERIALS AND METHODS

### 2.1 PET data acquisition

The study subjects were retrieved from the AIVO neuroinformatics project (http://aivo.utu.fi), in vivo molecular brain scan database hosted by Turku PET Centre. We identified all F18‐FMPEP‐d2 baseline PET studies of subjects without neurologic and psychiatric disorders, and current use of medications that could affect CNS or abuse of alcohol, nicotine or illicit drugs. Final sample consisted of 36 subjects of males with a mean age of 25.9 years (range, 21–35 years). All F18‐FMPEP‐d2 scans were acquired with GE Discovery VCT PET/computed tomography (CT) (GE Healthcare) between May 2017 and May 2019. PET scans were acquired for 60 min after injection of the tracer into subject's antecubital vein (the mean activity 187.9 MBq). CT scans were acquired before PET scans for attenuation correction. Magnetic resonance (MR) images (TR, 8.1 ms: TE, 3.7 ms: flip angle, 7°: scan time, 263 s: 1 mm^3^ isotropic voxels) were obtained with PET/MR (Ingenuity TF PET/MR, Philips) for anatomical normalization. The study was conducted in accordance with the Declaration of Helsinki and approved by the Turku University Hospital Clinical Research Services. The participants in this study were included in previous studies on feeding behavior and obesity (Kantonen, Karjalainen, et al., 2021; Kantonen, Pekkarinen, et al., 2021). PET images were processed with automated processing tool Magia (https://github.com/tkkarjal/magia) (Karjalainen et al., 2020). Magia uses FreeSurfer (http://surfer.nmr.mgh.harvard.edu/) to define the regions of interest (ROIs). As there exists no suitable central reference region for F18‐FMPEP‐d2, volume of distribution (*V*_*T*_) (Terry et al., 2010), FMPEP‐d2 *V*_*T*_ was quantified using graphical analysis by Logan (2000). The frames starting 36 min and more after injection were used in the model fitting, since Logan plots became linear after 36 min (Logan, 2000). Plasma activities were corrected for plasma metabolites as described previously (Lahesmaa et al., 2018). From F18‐FMPEP‐d2 PET scans, *V*_*T*_ from 21 ROIs were extracted: amygdala, hippocampus, caudate, putamen, nucleus accumbens, pallidum, thalamus, cerebellum, anterior cingulate cortex, posterior cingulate cortex, insula, orbitofrontal cortex, mid frontal cortex, superior temporal cortex, mid temporal cortex, inferior temporal cortex, superior frontal cortex, entorhinal cortex, midbrain, pons, and medulla.

### 2.2 Autoradiography

Autoradiography data were based on the study by Glass et al. with H3‐CP55940 (Glass et al., 1997). The data of H3‐CP55940 binding from 8 adult human brains with a mean age of 55.6 years (range, 21–81 years) were averaged across layers for each ROI and analyzed with mRNA expression of CNR1 gene. From the results of autoradiography, H3‐CP55940 binding was extracted from 15 ROIs: amygdala, hippocampus, caudate, putamen, nucleus accumbens, pallidum, thalamus, cerebellum, mid frontal cortex, mid temporal cortex, entorhinal cortex, cingulate, midbrain, pons, and medulla.

### 2.3 mRNA data

The gene expression information investigated in this study was obtained from the freely available Allen Human Brain Atlas (www.brain‐map.org) (Hawrylycz et al., 2012). We downloaded mRNA expressions of CNR1 gene (Cannabinoid receptor 1) to test for an association with the protein distribution. The dataset of the Allen Human Brain Atlas contains the gene expression profiles of CNR1 throughout the brain obtained from six healthy donors of 89 probes with a mean age of 42.5 years (range, 24–57 years). We downloaded mRNA expression of CNR1 gene in log2‐values for each sample. For ROI‐based analysis, mRNA expression values of CNR1 gene were obtained from the average within each region, and the median across probes (18 ROIs): amygdala, hippocampus, caudate, putamen, nucleus accumbens, thalamus, cerebellum, anterior cingulate cortex, posterior cingulate cortex, insula, orbitofrontal cortex, superior temporal cortex, mid temporal cortex, inferior temporal cortex, superior frontal cortex, midbrain, pons, and medulla.

### 2.4 Meta‐analysis

We performed a systematic search of MEDLINE (from inception to November 2021) for articles published in English using the keywords “positron emission tomography,” “single photon emission computed tomography”, “mRNA,” and “Allen human brain atlas.” All searches were limited to human studies. The inclusion criteria were primary studies that analyzed the association between protein expression from PET/single photon emission computed tomography (SPECT) and mRNA expression from the Allen Human Brain Atlas. If there was more than one published study using the same dataset of PET scans, only one report with the information most relevant to this study was included. Two authors performed the searches, screening independently, and discrepancies were resolved by consensus. Data were extracted from the publications independently by two reviewers, and the following information was recorded: first author, year of publication, ROIs, the number of patients, criteria for inclusion/exclusion, mRNA, and radiopharmaceuticals. Effect size was the correlation coefficient with 95% confidence interval (CI). Heterogeneity among studies was assessed using Cochran's *Q* and *I* ^2^ statistics, as described previously (Higgins et al., 2003). The pooled proportions are presented with random‐effects model. Meta‐analysis was performed using metafor package in R Statistical Software (version R 4.1.1, The R Foundation for Statistical Computing). The electronic search identified 164 articles from MEDLINE. We excluded conference abstracts, animal studies, non‐English studies, and studies that did not meet the inclusion criteria after screening the title or abstract. In addition, we included one result with the strongest correlation between each radiopharmaceutical from single institution with one mRNA expression, as several studies report the association between a single radiopharmaceutical with several mRNAs (C11‐Diprenorphine PET with OPRD1, OPRM1, OPRK1 (Rizzo et al., 2014): C11‐Flumazenil PET with GABRG1, GABRG2, GABRG3 GABRD, GABRB1, GABRB2, GABRB3, GABRA1, GABRA2, GABRA3, GABRA4, GABRA5, GABRA6, GABRQ (Nørgaard et al., 2021): C11‐PHNO PET with DRD2, DRD3 (Komorowski et al., 2020)). Also, we carefully evaluated the methods section of previous studies not to include the same dataset. If no indication of reuse was found, studies were considered as independent and the conventional random‐effects meta‐analysis assuming independence between studies is well suited for the statistical analysis. Finally, 11 studies were eligible for inclusion in this study (Beliveau et al., 2017; Kim et al., 2020; Komorowski et al., 2017, 2020; Lohith et al., 2017; Nørgaard et al., 2021; Pak et al., 2022; Rizzo et al., 2014, 2016; Veronese et al., 2016; Zanotti‐Fregonara et al., 2014).

### 2.5 Statistical analysis

Normality was assessed using the Shapiro–Wilks test. For the correlation analysis, Pearson correlation coefficient was calculated if the data were normally distributed. Otherwise, Spearman correlation coefficient was calculated. Autocorrelation of *V*_*T*_ from F18‐FMPEP‐d2 PET scans and CNR1 gene expression was assessed with Spearman correlation. For each ROI, mean *V*_*T*_ from F18‐FMPEP‐d2 PET scans, CNR1 gene expression, and H3‐CP55940 binding was calculated and Spearman or Pearson correlation analysis was performed to assess the association between them. To calculate a density map, the association between *V*_*T*_ from F18‐FMPEP‐d2 PET scans and H3‐CP55940 binding was estimated using a linear regression without intercept, and the estimated slopes were used to transform *V*_*T*_ into density values (Beliveau et al., 2017). Densities in units of femtomole per gram of tissue were converted to picomoles per millilitre using an approximate gray matter density of 1.045 g/ml (DiResta et al., 1991). All analyses were conducted using R Statistical Software (version R 4.1.1, The R Foundation for Statistical Computing).

## 3 RESULTS

**FIGURE 1:**
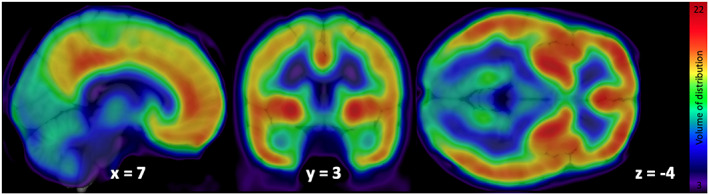
Mean volume of distribution of F18‐FMPEP‐d2 PET scans from 36 subjects.

**FIGURE 2:**
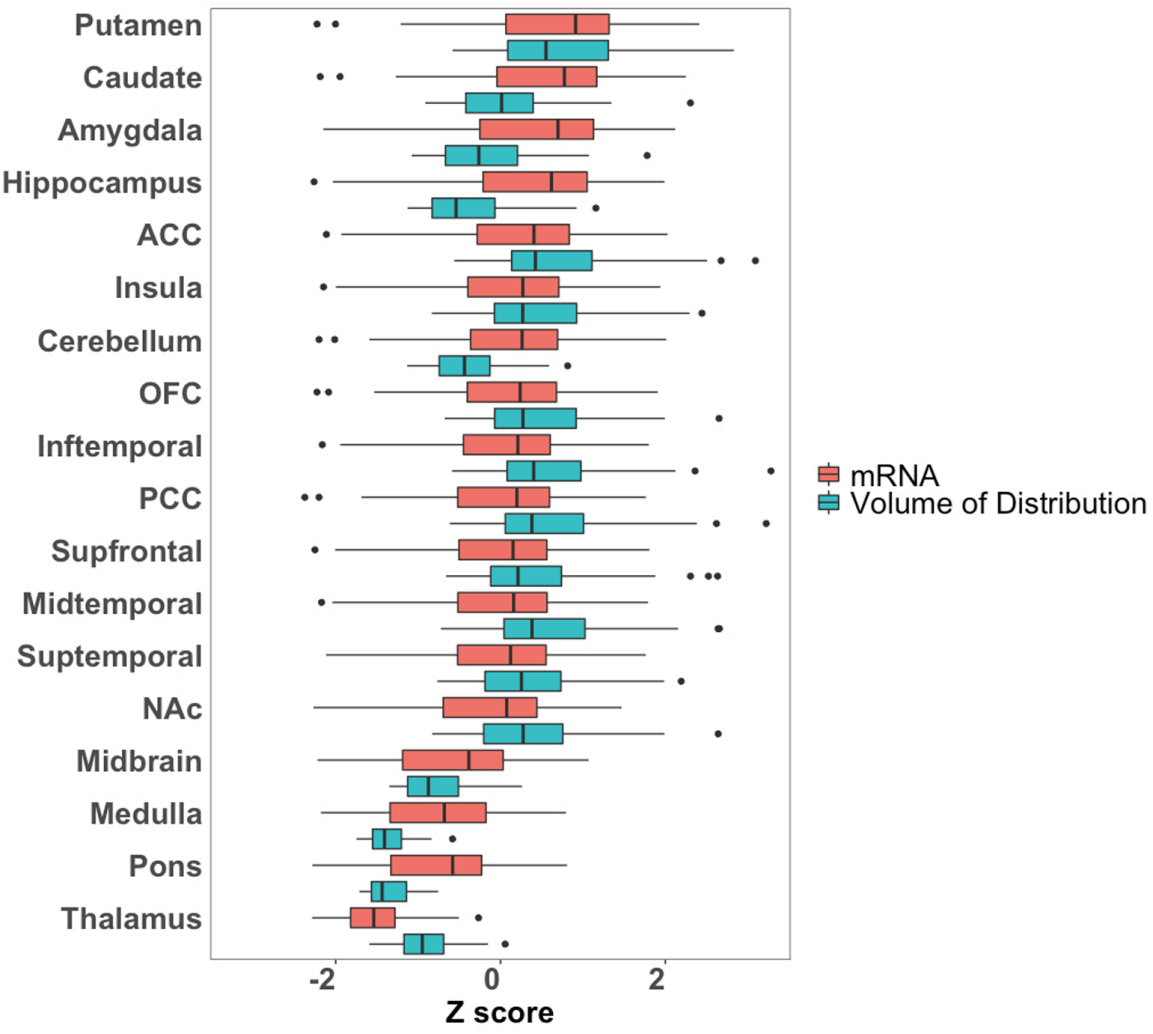
The distribution of CNR1 mRNA expression and volume of distribution of F18‐FMPEP‐d2 PET from 18 regions of interest. ACC, anterior cingulate cortex; NAc, nucleus accumbens; OFC, orbitofrontal cortex; PCC, posterior cingulate cortex.

**FIGURE 3:**
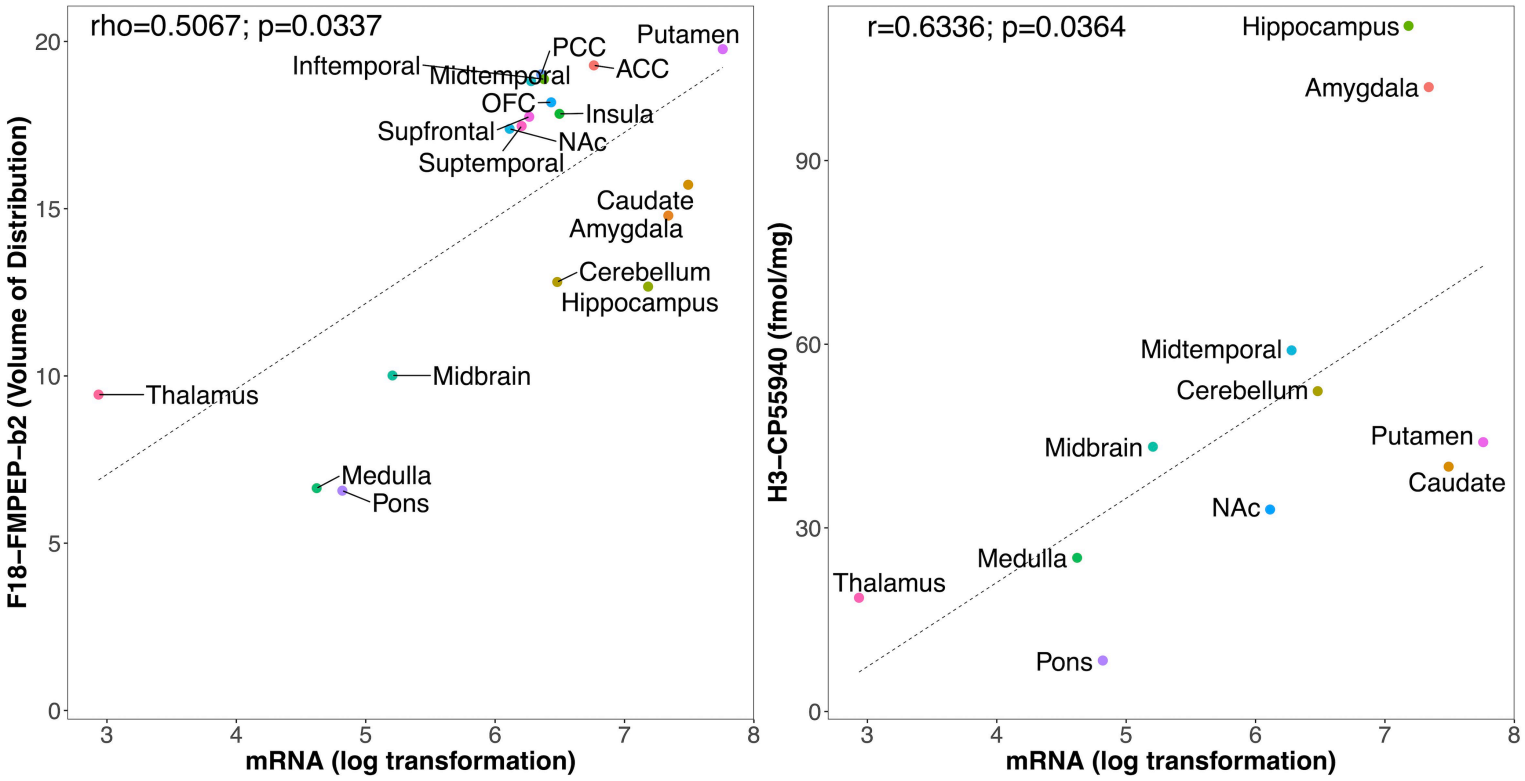
The association between CNR1 gene expression from the Allen human brain atlas and CB1 receptor expression. Left: Between volume of distribution of F18‐FMPEP‐d2 PET scans and CNR1 mRNA expression from 18 regions of interest, moderate strength of correlation was observed (*rho* = .5067, *p* = .0337). Right: Between H3‐CP55940 binding of autoradiography and CNR1 mRNA expression from 11 regions of interest, strong positive correlation was observed (*r* = .6336, *p* = .0364).

**FIGURE 4:**
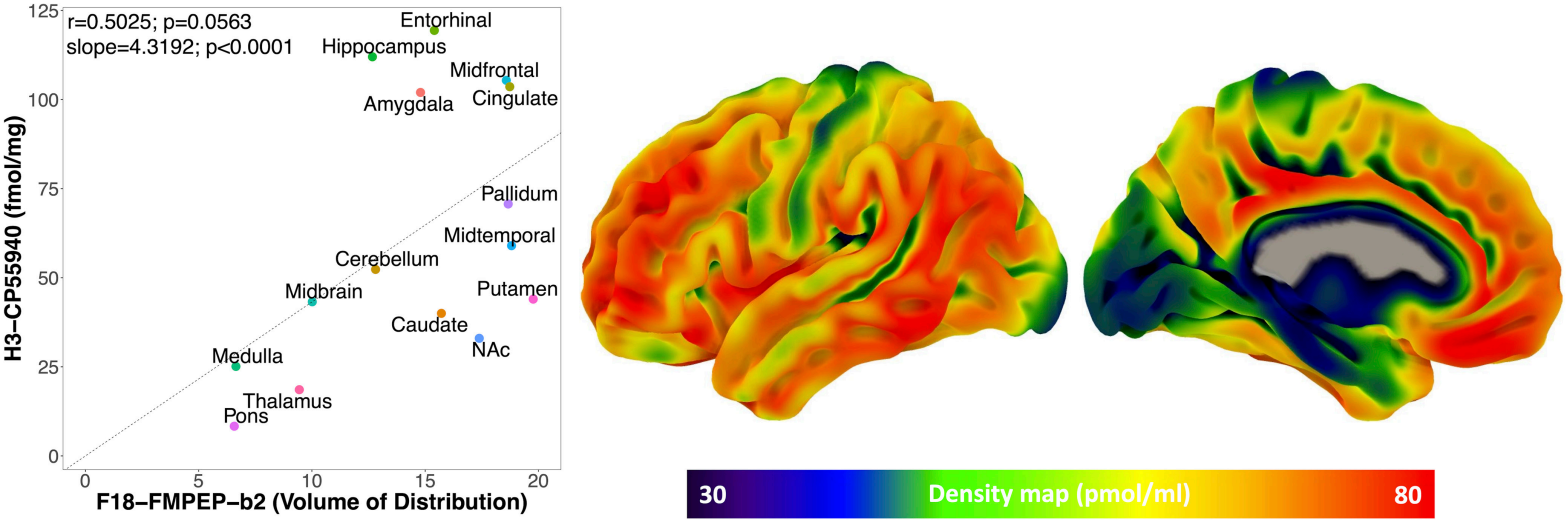
Left: Between volume of distribution of F18‐FMPEP‐d2 PET scans and H3‐CP55940 binding of autoradiography from 15 regions of interest, the correlation was not significant (*r* = .5025, *p* = .0563). The regressions line is fixed through 0 (the slope estimates = 4.3192; *p* < .0001). Right: Density map for CB1 receptor expression.

Mean distribution of F18‐FMPEP‐d2 PET scans from 36 subjects is visualized in Figure 1. The distribution of CNR1 mRNA expression and *V*_*T*_ of F18‐FMPEP‐d2 PET from each ROI is shown in Figure 2. Strong auto‐correlation was observed both for *V*_*T*_ from F18‐FMPEP‐d2 PET scans (inter‐subject: mean correlation coefficient *rho*: .8674) and for CNR1 mRNA expression from the Allen Human Brain Atlas (inter‐probe: mean correlation coefficient *rho* = .9148), which ensures consistency of observations. Between *V*_*T*_ of F18‐FMPEP‐d2 PET scans and CNR1 mRNA expression from 18 ROIs, moderate strength of correlation was observed (*rho* = .5067, *p* = .0337). The correlation between *V*_*T*_ of F18‐FMPEP‐d2 PET scans and mRNA was significant in subcortical regions (rho = .8182, *p* = .0068), not in cortical regions (rho = .5952, *p* = .1323). Strong positive correlation was also found between CNR1 mRNA expression and H3‐CP55940 binding from 11 ROIs (*r* = .6336, *p* = .0364), which validates the finding between F18‐FMPEP‐d2 PET scans and CNR1 mRNA (Figure 3). The correlation between *V*_*T*_ of F18‐FMPEP‐d2 PET scans and H3‐CP55940 binding from 15 ROIs was not significant (*r =* .5025, *p* = .0563). The slope estimates (slope = 4.3192; *p* < .0001) of the regression were used to transform F18‐FMPEP‐d2 PET atlas into density map atlas (Figure 4, https://neurovault.org/images/782713/).

**FIGURE 5:**
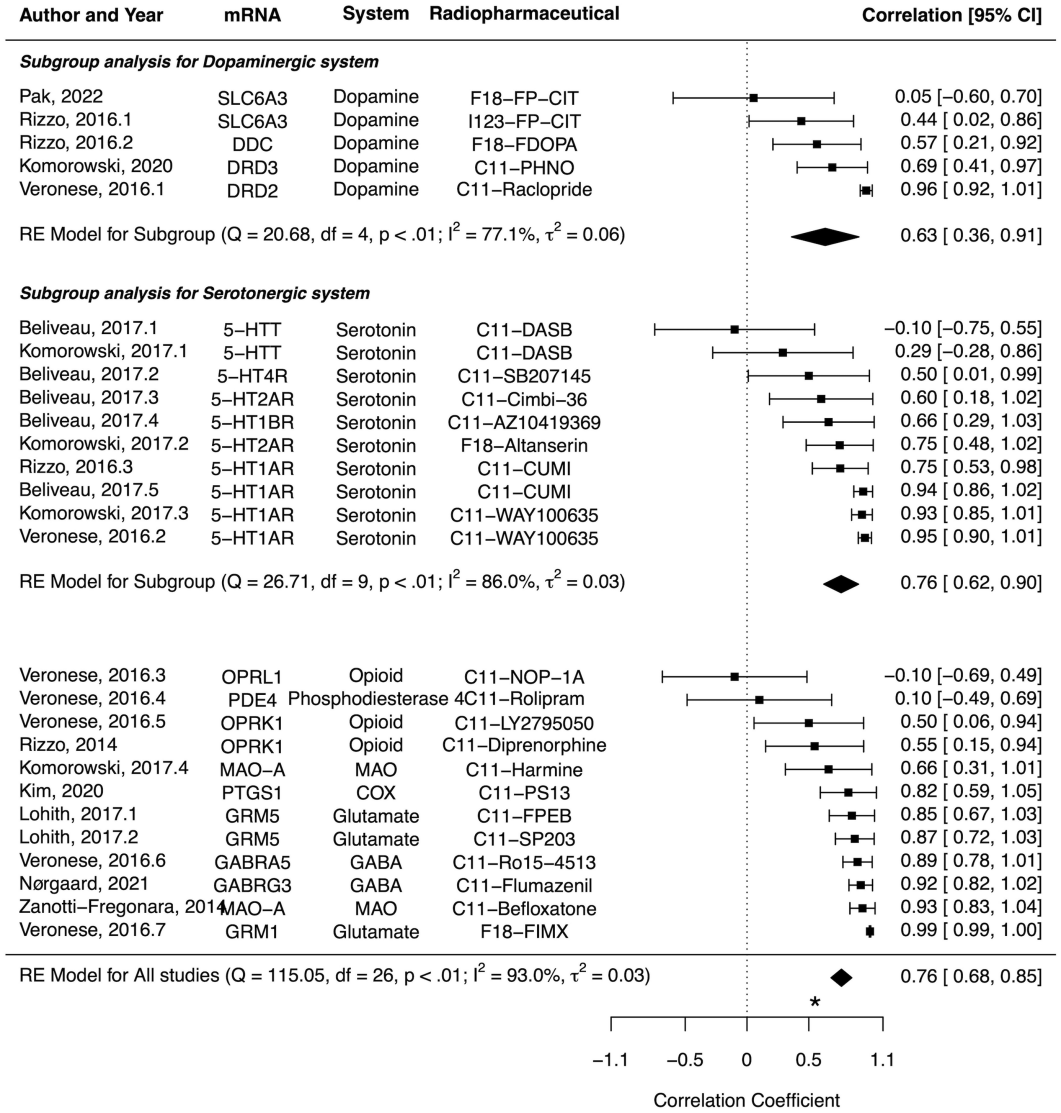
Forest plots for the correlation coefficient between protein expression from PET/single photon emission computed tomography (SPECT) and mRNA expression from the Allen human brain atlas. The pooled correlation coefficient was .76 (95% CI: .68–.85, *I* ^2^ = 93.0%). In a subgroup analysis, the pooled correlation coefficients were .63 (.36–.91, *I* ^2^ = 77.1%) for dopaminergic system, and .76 (.62–.90, *I* ^2^ = 86.0%) for serotonergic system, without a significant difference (*p* = .4099). *Asterisk indicates the correlation coefficient between CNR1 mRNA expression and CB1 receptor PET in this study.

The characteristics of studies included in the meta‐analysis are summarized in Table 1. The correlation coefficient ranged from −.10 between 5‐HTT mRNA and C11‐DASB PET to .99 between GRM1 mRNA and F18‐FIMX PET, with a pooled effect of .76 (95% CI: .68–.85, *I* ^2^ = 93.0%) (Figure 5). In a subgroup analysis, the pooled correlation coefficients were .63 (.36–.91, *I* ^2^ = 77.1%) for dopaminergic system, and .76 (.62–.90, *I* ^2^ = 86.0%) for serotonergic system, without a significant difference (*p* = .4099).

**TABLE 1:** Studies included in meta‐analysis

Author, year	Radiopharmaceutical	mRNA	System	Institution	No. of subjects
Rizzo et al. (2014)	C11‐Diprenorphine	OPRK1	Opioid	Hammersmith Hospital, London, UK	5
Zanotti‐Fregonara et al. (2014)	C11‐Befloxatone	MAOA	MAO		7
	C11‐Befloxatone	MAOA	MAO		8
Rizzo et al. (2016)	C11‐CUMI	HTR1A	Serotonin	Hammersmith Hospital, London, UK	13
	I123‐FP‐CIT	SLC6A3	Dopamine	Hospital Universitario Virgen del Rocío, Spain	
	F18‐FDOPA	DDC	Dopamine	Spain	
Veronese et al. (2016)	C11‐Raclopride	DRD2	Dopamine	Hammersmith Hospital, London, UK	10
	C11‐Ro15‐4513	GABRA5	GABA	Hammersmith Hospital, London, UK	4
	F18‐FIMX	GRM1	Glutamate	National Institutes of Health, USA	12
	C11‐NOP‐1A	OPRL1	Opioid	National Institutes of Health, USA	11
	C11‐LY2795050	OPRK1	Opioid	Yale PET Centre, USA	16
	C11‐WAY100635	HTR1A	Serotonin	Hammersmith Hospital, London, UK	15
	C11‐Rolipram	PDE4	Phosphodiesterase	National Institutes of Health, USA	12
Beliveau et al. (2017)	C11‐CUMI‐101	HTR1A	Serotonin	Cimbi database, Denmark	8
	C11‐AZ10419369	HTR1B	Serotonin	Cimbi database, Denmark	36
	C11‐Cimbi‐36	HTR2A	Serotonin	Cimbi database, Denmark	29
	C11‐SB207145	HTR4	Serotonin	Cimbi database, Denmark	59
	C11‐DASB	SLC6A4	Dopamine	Cimbi database, Denmark	100
Komorowski et al. (2017)	F18‐Altanserin	HTR2A	Serotonin	Research Center Juelich, Germany	19
	C11‐DASB	SLC6A4	Serotonin	Medical university of Vienna, Austria	34
	C11‐WAY100635	HTR1A	Serotonin	Medical university of Vienna, Austria	37
	C11‐Harmine	MAOA	MAO	Medical university of Vienna, Austria	22
Lohith et al. (2017)	C11‐SP203	GRM5	Glutamate	National Institutes of Health, USA	6
	C11‐FPEB	GRM5	Glutamate	National Institutes of Health, USA	8
Komorowski et al. (2020)	C11‐PHNO	DRD3	Dopamine	Medical university of Vienna, Austria	34
Kim et al. (2020)	C11‐PS13	COX1	COX	National Institutes of Health, USA	10
Nørgaard et al. (2021)	C11‐Flumazenil	GABRG3	GABA	Copenhagen University Hospital, Denmark	16
Pak et al. (2022)	F18‐FP‐CIT	SLC6A3	Dopamine	Pusan National University Hospital, Korea	35

Abbreviations: COX, cyclooxygenase; GABA, gamma‐aminobutyric acid; MAO, monoamine oxidase; mGluR5, metabotropic glutamate receptor 5.

## 4 DISCUSSION

Our main finding was that CNR1 mRNA expression was moderate to strongly correlated with CB1 receptor availability from *V*_*T*_ of F18‐FMPEP‐d2 PET, showing the association of CNR1 mRNA expression with CB1 receptor. The correlation between *V*_*T*_ of F18‐FMPEP‐d2 PET scans and H3‐CP55940 binding was marginally significant, and density map atlas of CB1 receptor was produced. From the meta‐analysis, the moderate to strong correlation was observed between mRNA expression from the Allen Human Brain Atlas and protein expressions from PET/SPECT scans across multiple genes, with the pooled correlation coefficient of .76, which was stronger than the correlation coefficient of .5067 between F18‐FMPEP‐d2 PET scans and CNR1 mRNA in this study.

The Allen Human Brain Atlas is a multimodal atlas of gene expression and anatomy of all brain regions from six postmortem brains with microarray‐based expression, in situ hybridization gene expression, and MR imaging‐based brain mapping (Shen et al., 2012). As the Allen Human Brain Atlas provides spatial expression profiles of multiple genes, the correlation study directly shows the predictive power of genes for in vivo protein levels from PET scans. In this regard, to understand brain function of CB1 receptor at the level of both mRNA and protein, the integration of brain mRNA atlas with PET scans and autoradiography was done in this study. These data show that the CB1 expression estimates derived from the radioligand F18‐FMPEP‐d2 are well in line with those from the ex vivo samples from the Allen Human Brain atlas, providing further evidence for the validity of this radioligand for mapping the endocannabinoid system.

The use of cannabinoids is of growing interest since several studies supported a great variety of pharmacological properties that would be useful in different pathologies, including neurological and psychiatric disorders (Black et al., 2019). THC derivatives dronabinol and nabilone are the first cannabinoids approved by FDA for the treatment of acquired immunodeficiency syndrome‐induced anorexia (dronabinol) and chemotherapy‐induced nausea and vomiting (dronabinol and nabilone) in 1985 (Khalsa et al., 2022). Also, CBD was approved in 2018 for treating seizures associated with Lennox–Gastaut syndrome or Dravet syndrome (Khalsa et al., 2022). An additional cannabis‐based drug is nabiximols, a combination of THC and CBD, which was approved for the treatment of spasticity in multiple sclerosis (Khalsa et al., 2022). CB1 receptor is a key component of the endocannabinoid system, which consists of cannabinoid receptors, endogenous ligands, and their metabolic enzymes (Tao et al., 2020). CB1 receptor, encoded by CNR1 gene, is expressed in cortex, hippocampus, amygdala, basal ganglia, substantia nigra, and cerebellum (Gifford et al., 2002; Herkenham et al., 1990; Mackie, 2005; Westlake et al., 1994) (Figures 1 and 2), and these circuits may be responsible for the behavioral effects of cannabis (Mackie, 2005). CB1 receptors are found primarily in the presynapses of the neurons, unlike other receptors of neurotransmitters which are located in the postsynapses (Mechoulam & Parker, 2013). Activation of CB1 receptors leads to a decrease in cyclic adenosine monophosphate accumulation (cAMP), inhibition of cAMP‐dependent protein kinase, and stimulation of mitogen‐activated protein kinase activity. CB1 receptor level is increased from adolescence to adulthood in rats, showing its importance in neurogenesis (Aguado et al., 2006; Verdurand et al., 2011). Also, alterations of CB1 receptor availability have been reported in neuropsychiatry disorders, such as post‐traumatic stress disorder and schizophrenia (Neumeister et al., 2013; Ranganathan et al., 2016; Volk et al., 2014). Therefore, CB1 receptor has become a target for drug development and in vivo imaging biomarker for neuropsychiatric disorders (Van Laere, 2007).

The association between CNR1 mRNA expression and protein expression from either PET scans or autoradiography showed the moderate to strong correlation, despite samples stemming from unrelated populations. However, we also have to consider that there are many complex and various post‐transcriptional mechanisms that are involved in turning mRNA into protein (Rizzo et al., 2016). mRNA transcripts interact with intra, extracellular stimuli, and are modified, regulated by non‐coding RNAs (Di Liegro et al., 2014), which have an influence on protein expression for each cell type (Cheng et al., 2005; Rizzo et al., 2014). In addition, technologies regarding measurement of either mRNA or protein expression may not be perfectly accurate (Veronese et al., 2016). Calculation of mRNA expression for each probe has its advantages and disadvantages (Arnatkeviciute et al., 2019). Therefore, probe selection has an impact on the final results of mRNA expression (Arnatkeviciute et al., 2019). In this study, we selected the median gene expression within each ROI to minimize the possible bias from probes. Also, mRNA expression is analyzed in the cytoplasm, while CB1 receptor is predominantly expressed in the cell membrane, presynaptically (Mechoulam & Parker, 2013). However, genomic atlas does not provide an accurate mapping at a cellular level, and mRNA expression as well as protein expressions from PET scans and autoradiography are averaged within each ROI, yielding representative expression values. Also, autoradiography provides far less spatial information than other technologies (Beliveau et al., 2017) and can be acquired only from postmortem states. These differences in PET scans and autoradiography might affect non‐significant association between *V*_*T*_ of F18‐FMPEP‐d2 PET scans and H3‐CP55940 binding.

Previously, the association of brain mRNA mappings of the Allen Human Brain Atlas with several PET‐derived protein expressions has been investigated, including serotonin receptors (Beliveau et al., 2017; Komorowski et al., 2017; Rizzo et al., 2014), serotonin transporters (Beliveau et al., 2017; Komorowski et al., 2017), opioid receptors (Rizzo et al., 2014), dopamine receptors (Komorowski et al., 2020), and monoamine oxidase A (MAO‐A) (Komorowski et al., 2017; Zanotti‐Fregonara et al., 2014). The correlation coefficient ranged from −.10 between 5‐HTT mRNA and C11‐DASB PET to .99 between GRM1 mRNA and F18‐FIMX PET. We included one result with the strongest correlation between each radiopharmaceutical from single institution with one mRNA expression, as several studies report the association between a single radiopharmaceutical with several mRNAs. The presently observed correlations for CB1 (*rho* = .5067, *p* = .0337) was slightly weaker than the pooled result of this meta‐analysis. Even in the same neurotransmitter system, there was a difference in the associations between mRNA expression and protein expression according to the receptor or transporter. With the same gene expression of HTR1A mRNA from the Allen Human Brain Atlas, a wide range of correlation coefficients with protein expression has been shown; .75–.95, probably due to the characteristics of radiopharmaceuticals and ROIs included in each study. In addition, the majority of the studies included only a small number of subjects, typically <30. However, the moderate to strong correlation was observed between mRNA expression and protein expressions across multiple genes, showing the association of genes with protein levels of human brains. Recently, Hansen et al. reported the correlation between mRNA expression and multiple neurotransmitter receptors and transporters including CB1 receptor (Justine et al., 2022). Poor spatial correspondences between mRNA expression and protein expression from PET scans were observed, except for 5 metabotropic receptors including CB1 receptor with a correlation coefficient of .66 (Justine et al., 2022). However, their report did not include the relevant autoradiographic finding. In addition, subjects were scanned with the different radiopharmaceutical of C11‐OMAR with this study, making it difficult to compare with our result. There are a number of limitations that should be taken into account. First, F18‐FMPEP‐d2 PET scans were acquired from young males included in previous studies on feeding behavior and obesity (Kantonen, Karjalainen, et al., 2021; Kantonen, Pekkarinen, et al., 2021). Although no effect of age on CB1 receptor was reported in previous studies (Borgan et al., 2019; Hirvonen et al., 2012, 2013), there is a difference in the age of participants between PET scan, autoradiography, and Allen Human Brain Atlas. The sample size of Allen Human Brain Atlas (*n* = 6) is too small to evaluate the age effect on mRNA expression and to give accurate result of the degree of correlation. In addition, the autoradiography study (Glass et al., 1997) provides the average density of H3‐CP55940 binding in the adult human brain, without the density of each subject. Therefore, age could not be included as a covariate in this study. Second, while making the density atlas map of CB1 receptor, an area devoid of CB1 receptor would be estimated to have positive value of CB1 receptor density. A more accurate estimation for the linear conversion of *V*_*T*_ to receptor density would be set the intercept = volume of non‐displaceable uptake. The linear conversion to receptor density will be erroneous for small *V*_*T*_. However, we used *V*_*T*_ as the outcome measure to keep the results comparable with previous reports on the same CB1 receptor dataset (Kantonen, Karjalainen, et al., 2021; Kantonen, Pekkarinen, et al., 2021). Third, the method of calculation of mRNA expression from the Allen Human Brain Atlas and *V*_*T*_ of F18‐FMPEP‐d2 PET might have an impact on the final results of this study. Further studies are needed to examine the association between protein expression based on multiple PET radioligands and gene expression with a uniform method.

In conclusion, we observed the moderate to strong associations between gene and protein expression for CB1 receptor in the human brain. Also, this association between CNR1 mRNA and CB1 receptor protein expression was validated by autoradiography. We combined the autoradiographic finding with PET of CB1 receptor, producing the density atlas map of CB1 receptor for the first time in the human brain. There have been multiple studies investigating the association between mRNA and protein expression in the human brain, showing the heterogeneous results; however, the moderate to strong correlation was observed between mRNA expression and protein expressions across multiple genes, showing the significant association of genes with protein levels. Further study is needed to investigate the relationship between multiple genes and in vivo proteins to improve and accelerate drug development.

### DECLARATION OF TRANSPARENCY

The authors, reviewers and editors affirm that in accordance to the policies set by the *Journal of Neuroscience Research*, this manuscript presents an accurate and transparent account of the study being reported and that all critical details describing the methods and results are present.

## AUTHOR CONTRIBUTIONS

*Conceptualization*, K.P., T.K., L.N.; *Methodology*, K.P., T.K., L.N.; *Software*, K.P., T.K., L.P.; *Investigation*, K.P., T.K., L.P., L.N.; *Writing – Original Draft*, K.P., P.N., L.N.; *Supervision*, K.P., P.N., L.N.

## FUNDING INFORMATION

This study was supported by the Academy of Finland (grants # 332225) and National Research Foundation of Korea (2020R1F1A1054201).

## CONFLICT OF INTEREST

The authors declare no competing interests.

### PEER REVIEW

The peer review history for this article is available at https://publons.com/publon/10.1002/jnr.25149.

## Supporting information

Transparent Science Questionnaire for Authors

## Data Availability

Data will be available from the corresponding author on reasonable request.
